# 3-Benzyl-2-phenyl-1,3-thia­zolidin-4-one

**DOI:** 10.1107/S1600536811037706

**Published:** 2011-09-30

**Authors:** Hoong-Kun Fun, Madhukar Hemamalini, Poovan Shanmugavelan, Alagusundaram Ponnuswamy, Rathinavel Jagatheesan

**Affiliations:** a X-ray Crystallography Unit, School of Physics, Universiti Sains Malaysia, 11800 USM, Penang, Malaysia; b Department of Organic Chemistry, School of Chemistry, Madurai Kamaraj University, Madurai 625 021, Tamil Nadu, India; c Department of Chemistry, Thanthai Hans Roever College, Perambalur 621 212, Tamil Nadu, India

## Abstract

In the title compound, C_16_H_15_NOS, the thia­zolidine ring, which is essentially planar [maximum deviation = 0.071 (2) Å], makes dihedral angles of 88.01 (8) and 87.21 (8)° with the terminal phenyl rings. The dihedral angle between the phenyl rings is 49.45 (5)°. In the crystal, mol­ecules are linked by a weak inter­molecular C—H⋯O hydrogen bond, forming a supra­molecular chain along the *b* axis. Furthermore, the crystal packing is stabilized by a weak C—H⋯π inter­action.

## Related literature

For details and applications of thia­zolidine-4-ones, see: Dutta *et al.* (1990 ▶); Jadhav & Ingle (1978 ▶); Gursoy & Terzioglu (2005 ▶); Rawal *et al.* (2007 ▶); Shrivastava *et al.* (2005 ▶); Look *et al.* (1996 ▶); Anders *et al.* (2001 ▶); Barreca *et al.* (2001 ▶); Diurno *et al.* (1992 ▶).
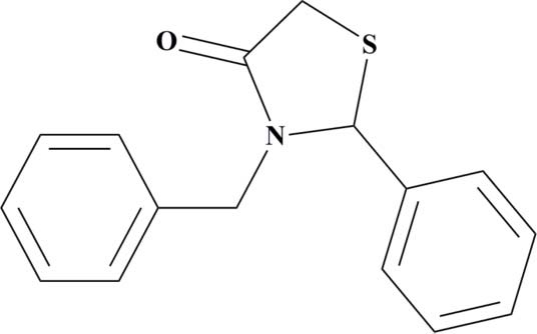


## Experimental

### 

#### Crystal data

C_16_H_15_NOS
*M* *_r_* = 269.35
Monoclinic, 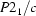

*a* = 13.5734 (15) Å
*b* = 10.1402 (11) Å
*c* = 10.1496 (11) Å
β = 104.305 (2)°
*V* = 1353.6 (3) Å^3^
*Z* = 4
Mo *K*α radiation
μ = 0.23 mm^−1^
*T* = 296 K
0.41 × 0.19 × 0.06 mm

#### Data collection

Bruker APEXII DUO CCD area-detector diffractometer
Absorption correction: multi-scan (*SADABS*; Bruker, 2009 ▶) *T*_min_ = 0.913, *T*_max_ = 0.985
21164 measured reflections
3990 independent reflections
2813 reflections with *I* > 2σ(*I*)
*R*_int_ = 0.041

#### Refinement

*R*[*F* ^2^ > 2σ(*F* ^2^)] = 0.040
*wR*(*F* ^2^) = 0.112
*S* = 1.05
3990 reflections
172 parameters
H-atom parameters constrained
Δρ_max_ = 0.21 e Å^−3^
Δρ_min_ = −0.25 e Å^−3^

### 

Data collection: *APEX2* (Bruker, 2009 ▶); cell refinement: *SAINT* (Bruker, 2009 ▶); data reduction: *SAINT*; program(s) used to solve structure: *SHELXTL* (Sheldrick, 2008 ▶); program(s) used to refine structure: *SHELXTL*; molecular graphics: *SHELXTL*; software used to prepare material for publication: *SHELXTL* and *PLATON* (Spek, 2009 ▶).

## Supplementary Material

Crystal structure: contains datablock(s) global, I. DOI: 10.1107/S1600536811037706/is2778sup1.cif

Structure factors: contains datablock(s) I. DOI: 10.1107/S1600536811037706/is2778Isup2.hkl

Supplementary material file. DOI: 10.1107/S1600536811037706/is2778Isup3.cml

Additional supplementary materials:  crystallographic information; 3D view; checkCIF report

## Figures and Tables

**Table 1:** Hydrogen-bond geometry (Å, °) *Cg*1 is the centroid of the C11–C16 ring.

*D*—H⋯*A*	*D*—H	H⋯*A*	*D*⋯*A*	*D*—H⋯*A*
C14—H14*A*⋯O1^i^	0.93	2.47	3.323 (2)	153
C2—H2*A*⋯*Cg*1^ii^	0.93	2.99	3.705 (3)	134

Symmetry codes: (i) 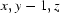; (ii) 
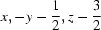
.

**Fig. 1.**
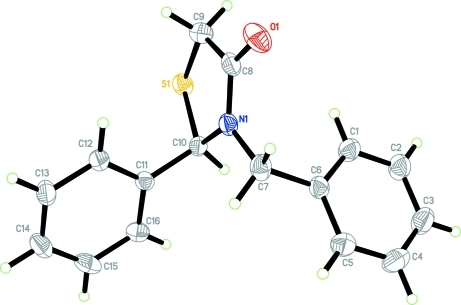
An ORTEP view of the title compound, showing 30% probability displacement ellipsoids.

**Fig. 2.**
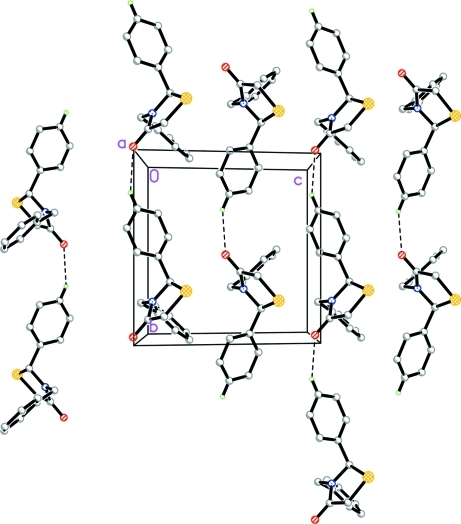
The crystal packing of the title compound, viewed along the a axis.

## supplementary crystallographic information

### Comment

One of the main objectives of organic and medicinal chemistry is the design, synthesis and production of molecules having value as human therapeutic agents. During the past decade, combinatorial chemistry has provided access to chemical libraries based on privileged structures with heterocyclic moiety receiving special attention as they belong to a class of compounds with proven utility in medicinal chemistry.  There are numerous biologically active molecules with five-membered rings, containing two hetero atoms. Among them, thiazolidin-4-ones are the most extensively investigated class of compounds, which have many interesting activity profiles namely bactericidal (Dutta *et al.*, 1990), antifungal (Jadhav & Ingle, 1978), anticonvulsant (Gursoy & Terzioglu, 2005), anti-HIV (Rawal *et al.*, 2007), antituberculotic (Shrivastava *et al.*, 2005), COX-1 inhibitors (Look *et al.*, 1996), inhibitors of the bacterial enzyme MurB (Anders *et al.*, 2001), non-nucleoside inhibitors of HIV-RT (Barreca *et al.*, 2001) and anti-histaminic agents (Diurno *et al.*, 1992).

The asymmetric unit of the title compound is shown in Fig. 1.  The thiazolidine (S1/N1/C8–C10) ring is essentially planar, with a maximum deviation of 0.071 (2) Å for atom C10.  The central thiazolidine (S1/N1/C8–C10) ring makes dihedral angles of 88.01 (8) and 87.21 (8)° with the terminal phenyl (C1–C6) and (C11–C16) rings, respectively. The dihedral angle between the phenyl (C1–C6) and (C11–C16) rings is 49.45 (5)°.

In the crystal structure, (Fig. 2),  the molecules are linked by intermolecular weak C—H···O hydrogen bonds forming supramolecular chains along the *b*-axis.  Furthermore, the crystal packing is stabilized by weak C—H···π interactions involving the C11–C16 ring.

### Experimental

To a well ground intimate mixture of triphenyl phosphine (0.43 g, 1.6 mmol) and benzaldehyde, (0.15 g, 1.5 mmol) in a microwave vial (10 ml) equipped with a magnetic stirring bar, benzylazide, (0.2 g, 1.5 mmol) was added in drop with stirring.  Stirring was continued until liberation of nitrogen ceased and then mercaptoacetic acid, (0.15 g, 1.6 mmol) was added to the above mixture and the reaction vessel was sealed with a septum.  It was then placed into the cavity of a focused monomode microwave reactor (CEM Discover, benchmate) and operated at 150°C (temperature monitored by a built-in IR sensor), power 80W for 10 minutes.  The reaction temperature was maintained by modulating the power level of the reactor.  The reaction mixture was allowed to stand at room temperature.  Then the residue was purified by column chromatography on silica (petrolium ether–ethyl acetate, 94:6) to afford the 3-benzyl-2-phenylthiazolidin-4-one. Yield: 0.38g (95%); m.p. 152–155°C.

### Refinement

All hydrogen atoms were positioned geometrically (C—H = 0.93–0.98 Å) and were refined using a riding model, with *U*_iso_(H) = 1.2*U*_eq_(C).

### Crystal data

**Table:**

C_16_H_15_NOS	*F*(000) = 568
*M**_r_* = 269.35	*D*_x_ = 1.322 Mg m^−^^3^
Monoclinic, *P*2_1_/*c*	Mo *K*α radiation, λ = 0.71073 Å
Hall symbol: -P 2ybc	Cell parameters from 4044 reflections
*a* = 13.5734 (15) Å	θ = 2.9–24.9°
*b* = 10.1402 (11) Å	µ = 0.23 mm^−^^1^
*c* = 10.1496 (11) Å	*T* = 296 K
β = 104.305 (2)°	Plate, colourless
*V* = 1353.6 (3) Å^3^	0.41 × 0.19 × 0.06 mm
*Z* = 4	

### Data collection

**Table:**

Bruker APEXII DUO CCD area-detector diffractometer	3990 independent reflections
Radiation source: fine-focus sealed tube	2813 reflections with *I* > 2σ(*I*)
graphite	*R*_int_ = 0.041
φ and ω scans	θ_max_ = 30.2°, θ_min_ = 1.6°
Absorption correction: multi-scan (*SADABS*; Bruker, 2009)	*h* = −19→19
*T*_min_ = 0.913, *T*_max_ = 0.985	*k* = −14→14
21164 measured reflections	*l* = −14→14

### Refinement

**Table:**

Refinement on *F*^2^	Primary atom site location: structure-invariant direct methods
Least-squares matrix: full	Secondary atom site location: difference Fourier map
*R*[*F*^2^ > 2σ(*F*^2^)] = 0.040	Hydrogen site location: inferred from neighbouring sites
*wR*(*F*^2^) = 0.112	H-atom parameters constrained
*S* = 1.05	*w* = 1/[σ^2^(*F*_o_^2^) + (0.0448*P*)^2^ + 0.2789*P*] where *P* = (*F*_o_^2^ + 2*F*_c_^2^)/3
3990 reflections	(Δ/σ)_max_ = 0.001
172 parameters	Δρ_max_ = 0.21 e Å^−^^3^
0 restraints	Δρ_min_ = −0.25 e Å^−^^3^

### Special details

**Table:**

Geometry. All s.u.'s (except the s.u. in the dihedral angle between two l.s. planes) are estimated using the full covariance matrix. The cell s.u.'s are taken into account individually in the estimation of s.u.'s in distances, angles and torsion angles; correlations between s.u.'s in cell parameters are only used when they are defined by crystal symmetry. An approximate (isotropic) treatment of cell s.u.'s is used for estimating s.u.'s involving l.s. planes.
Refinement. Refinement of F^2^ against ALL reflections. The weighted R-factor wR and goodness of fit S are based on F^2^, conventional R-factors R are based on F, with F set to zero for negative F^2^. The threshold expression of F^2^ > 2σ(F^2^) is used only for calculating R-factors(gt) etc. and is not relevant to the choice of reflections for refinement. R-factors based on F^2^ are statistically about twice as large as those based on F, and R- factors based on ALL data will be even larger.

### Fractional atomic coordinates and isotropic or equivalent isotropic displacement parameters (Å^2^)

**Table:**

	*x*	*y*	*z*	*U*_iso_*/*U*_eq_	
S1	0.04954 (3)	0.22379 (4)	0.22886 (4)	0.04505 (12)	
O1	0.16669 (11)	0.46692 (11)	0.51751 (13)	0.0650 (4)	
N1	0.21308 (9)	0.28385 (11)	0.41853 (12)	0.0389 (3)	
C1	0.34627 (12)	0.44309 (17)	0.30014 (17)	0.0510 (4)	
H1A	0.2783	0.4676	0.2838	0.061*	
C2	0.40708 (14)	0.49962 (19)	0.22451 (19)	0.0582 (4)	
H2A	0.3798	0.5617	0.1580	0.070*	
C3	0.50706 (14)	0.4646 (2)	0.2471 (2)	0.0637 (5)	
H3A	0.5481	0.5024	0.1963	0.076*	
C4	0.54623 (15)	0.3732 (2)	0.3454 (3)	0.0810 (7)	
H4A	0.6143	0.3492	0.3613	0.097*	
C5	0.48589 (14)	0.3161 (2)	0.4212 (2)	0.0650 (5)	
H5A	0.5136	0.2538	0.4873	0.078*	
C6	0.38484 (11)	0.35067 (14)	0.39972 (15)	0.0410 (3)	
C7	0.32037 (12)	0.29270 (17)	0.48752 (16)	0.0479 (4)	
H7A	0.3454	0.2052	0.5166	0.057*	
H7B	0.3278	0.3467	0.5683	0.057*	
C8	0.14552 (12)	0.37589 (14)	0.43636 (15)	0.0434 (3)	
C9	0.04128 (13)	0.35455 (17)	0.34511 (19)	0.0533 (4)	
H9A	0.0173	0.4347	0.2953	0.064*	
H9B	−0.0061	0.3309	0.3987	0.064*	
C10	0.17936 (10)	0.17886 (13)	0.32051 (14)	0.0358 (3)	
H10A	0.2225	0.1788	0.2560	0.043*	
C11	0.18426 (10)	0.04347 (13)	0.38516 (13)	0.0350 (3)	
C12	0.14070 (11)	0.02045 (14)	0.49347 (15)	0.0420 (3)	
H12A	0.1104	0.0896	0.5291	0.050*	
C13	0.14219 (12)	−0.10439 (16)	0.54857 (17)	0.0499 (4)	
H13A	0.1127	−0.1188	0.6209	0.060*	
C14	0.18706 (13)	−0.20754 (16)	0.49698 (19)	0.0547 (4)	
H14A	0.1879	−0.2915	0.5342	0.066*	
C15	0.23077 (14)	−0.18557 (16)	0.38958 (19)	0.0561 (4)	
H15A	0.2611	−0.2550	0.3544	0.067*	
C16	0.22959 (12)	−0.06032 (15)	0.33392 (16)	0.0455 (4)	
H16A	0.2594	−0.0461	0.2619	0.055*	

### Atomic displacement parameters (Å^2^)

**Table:**

	*U*^11^	*U*^22^	*U*^33^	*U*^12^	*U*^13^	*U*^23^
S1	0.0441 (2)	0.0416 (2)	0.0451 (2)	−0.00081 (16)	0.00278 (16)	0.00479 (16)
O1	0.0919 (10)	0.0400 (6)	0.0633 (7)	−0.0033 (6)	0.0195 (7)	−0.0119 (6)
N1	0.0399 (6)	0.0325 (6)	0.0436 (6)	−0.0060 (5)	0.0088 (5)	−0.0002 (5)
C1	0.0404 (8)	0.0553 (10)	0.0561 (9)	0.0005 (7)	0.0099 (7)	0.0130 (7)
C2	0.0548 (10)	0.0599 (11)	0.0594 (10)	−0.0048 (8)	0.0132 (8)	0.0154 (8)
C3	0.0508 (10)	0.0721 (13)	0.0721 (12)	−0.0090 (9)	0.0225 (9)	0.0089 (10)
C4	0.0441 (10)	0.0872 (16)	0.1158 (18)	0.0105 (10)	0.0273 (11)	0.0296 (14)
C5	0.0480 (10)	0.0596 (11)	0.0852 (13)	0.0083 (8)	0.0119 (9)	0.0229 (10)
C6	0.0383 (7)	0.0363 (7)	0.0452 (8)	−0.0057 (6)	0.0041 (6)	−0.0011 (6)
C7	0.0456 (8)	0.0481 (9)	0.0450 (8)	−0.0090 (7)	0.0020 (7)	0.0074 (7)
C8	0.0561 (9)	0.0309 (7)	0.0462 (8)	−0.0036 (6)	0.0185 (7)	0.0023 (6)
C9	0.0496 (9)	0.0456 (9)	0.0662 (10)	0.0059 (7)	0.0173 (8)	−0.0016 (8)
C10	0.0372 (7)	0.0346 (7)	0.0369 (7)	−0.0029 (5)	0.0117 (5)	−0.0001 (5)
C11	0.0339 (7)	0.0319 (6)	0.0382 (7)	−0.0008 (5)	0.0069 (5)	−0.0012 (5)
C12	0.0450 (8)	0.0365 (7)	0.0472 (8)	−0.0005 (6)	0.0165 (6)	0.0010 (6)
C13	0.0495 (9)	0.0448 (9)	0.0555 (9)	−0.0074 (7)	0.0130 (7)	0.0118 (7)
C14	0.0547 (10)	0.0337 (8)	0.0667 (11)	−0.0038 (7)	−0.0020 (8)	0.0079 (7)
C15	0.0611 (11)	0.0368 (8)	0.0649 (11)	0.0126 (7)	0.0050 (9)	−0.0067 (7)
C16	0.0471 (8)	0.0443 (8)	0.0451 (8)	0.0074 (7)	0.0111 (7)	−0.0034 (6)

### Geometric parameters (Å, °)

**Table:**

S1—C9	1.7967 (17)	C7—H7A	0.9700
S1—C10	1.8352 (14)	C7—H7B	0.9700
O1—C8	1.2231 (18)	C8—C9	1.503 (2)
N1—C8	1.3515 (19)	C9—H9A	0.9700
N1—C10	1.4518 (18)	C9—H9B	0.9700
N1—C7	1.4544 (19)	C10—C11	1.5160 (19)
C1—C2	1.383 (2)	C10—H10A	0.9800
C1—C6	1.383 (2)	C11—C16	1.3838 (19)
C1—H1A	0.9300	C11—C12	1.391 (2)
C2—C3	1.366 (3)	C12—C13	1.382 (2)
C2—H2A	0.9300	C12—H12A	0.9300
C3—C4	1.369 (3)	C13—C14	1.377 (2)
C3—H3A	0.9300	C13—H13A	0.9300
C4—C5	1.382 (3)	C14—C15	1.382 (3)
C4—H4A	0.9300	C14—H14A	0.9300
C5—C6	1.380 (2)	C15—C16	1.389 (2)
C5—H5A	0.9300	C15—H15A	0.9300
C6—C7	1.513 (2)	C16—H16A	0.9300
			
C9—S1—C10	93.34 (7)	C8—C9—S1	107.99 (11)
C8—N1—C10	119.26 (12)	C8—C9—H9A	110.1
C8—N1—C7	121.65 (13)	S1—C9—H9A	110.1
C10—N1—C7	118.93 (12)	C8—C9—H9B	110.1
C2—C1—C6	121.10 (15)	S1—C9—H9B	110.1
C2—C1—H1A	119.4	H9A—C9—H9B	108.4
C6—C1—H1A	119.4	N1—C10—C11	113.25 (11)
C3—C2—C1	120.23 (17)	N1—C10—S1	105.43 (9)
C3—C2—H2A	119.9	C11—C10—S1	112.22 (9)
C1—C2—H2A	119.9	N1—C10—H10A	108.6
C2—C3—C4	119.27 (17)	C11—C10—H10A	108.6
C2—C3—H3A	120.4	S1—C10—H10A	108.6
C4—C3—H3A	120.4	C16—C11—C12	119.00 (13)
C3—C4—C5	120.83 (18)	C16—C11—C10	120.14 (13)
C3—C4—H4A	119.6	C12—C11—C10	120.83 (12)
C5—C4—H4A	119.6	C13—C12—C11	120.47 (14)
C6—C5—C4	120.54 (17)	C13—C12—H12A	119.8
C6—C5—H5A	119.7	C11—C12—H12A	119.8
C4—C5—H5A	119.7	C14—C13—C12	120.36 (16)
C5—C6—C1	118.02 (15)	C14—C13—H13A	119.8
C5—C6—C7	120.33 (14)	C12—C13—H13A	119.8
C1—C6—C7	121.60 (14)	C13—C14—C15	119.60 (15)
N1—C7—C6	113.35 (12)	C13—C14—H14A	120.2
N1—C7—H7A	108.9	C15—C14—H14A	120.2
C6—C7—H7A	108.9	C14—C15—C16	120.29 (15)
N1—C7—H7B	108.9	C14—C15—H15A	119.9
C6—C7—H7B	108.9	C16—C15—H15A	119.9
H7A—C7—H7B	107.7	C11—C16—C15	120.28 (15)
O1—C8—N1	123.85 (15)	C11—C16—H16A	119.9
O1—C8—C9	123.53 (15)	C15—C16—H16A	119.9
N1—C8—C9	112.62 (13)		
			
C6—C1—C2—C3	0.1 (3)	C8—N1—C10—C11	−114.56 (14)
C1—C2—C3—C4	−0.1 (3)	C7—N1—C10—C11	69.99 (16)
C2—C3—C4—C5	0.2 (4)	C8—N1—C10—S1	8.50 (15)
C3—C4—C5—C6	−0.3 (4)	C7—N1—C10—S1	−166.95 (10)
C4—C5—C6—C1	0.3 (3)	C9—S1—C10—N1	−10.43 (10)
C4—C5—C6—C7	−177.10 (19)	C9—S1—C10—C11	113.28 (11)
C2—C1—C6—C5	−0.2 (3)	N1—C10—C11—C16	−131.16 (14)
C2—C1—C6—C7	177.17 (16)	S1—C10—C11—C16	109.62 (13)
C8—N1—C7—C6	−98.30 (17)	N1—C10—C11—C12	50.93 (18)
C10—N1—C7—C6	77.04 (17)	S1—C10—C11—C12	−68.30 (15)
C5—C6—C7—N1	−151.78 (16)	C16—C11—C12—C13	−0.4 (2)
C1—C6—C7—N1	30.9 (2)	C10—C11—C12—C13	177.59 (13)
C10—N1—C8—O1	179.17 (14)	C11—C12—C13—C14	0.1 (2)
C7—N1—C8—O1	−5.5 (2)	C12—C13—C14—C15	0.0 (3)
C10—N1—C8—C9	−1.01 (18)	C13—C14—C15—C16	0.0 (3)
C7—N1—C8—C9	174.31 (13)	C12—C11—C16—C15	0.4 (2)
O1—C8—C9—S1	172.54 (13)	C10—C11—C16—C15	−177.53 (14)
N1—C8—C9—S1	−7.28 (16)	C14—C15—C16—C11	−0.3 (2)
C10—S1—C9—C8	10.22 (12)		

### Hydrogen-bond geometry (Å, °)

**Table:**

*Cg*1 is the centroid of the C11–C16 ring.

**Table:**

*D*—H···*A*	*D*—H	H···*A*	*D*···*A*	*D*—H···*A*
C14—H14A···O1^i^	0.93	2.47	3.323 (2)	153
C2—H2A···Cg1^ii^	0.93	2.99	3.705 (3)	134

Symmetry codes: (i) *x*, *y*−1, *z*; (ii) *x*, −*y*−1/2, *z*−3/2.
